# Men at Risk; a Qualitative Study on HIV Risk, Gender Identity and Violence among Men Who Have Sex with Men Who Report High Risk Behavior in Kampala, Uganda

**DOI:** 10.1371/journal.pone.0082937

**Published:** 2013-12-17

**Authors:** Rachel King, Joseph Barker, Sylvia Nakayiwa, David Katuntu, George Lubwama, Danstan Bagenda, Tim Lane, Alex Opio, Wolfgang Hladik

**Affiliations:** 1 Global Health Sciences, University of California, San Francisco, California, United States of America; 2 Division of Global HIV/AIDS, Center for Global Health, Centers for Disease Control and Prevention (CDC), Entebbe, Uganda; 3 Makerere University School of Public Health (MakSPH), Kampala, Uganda; 4 Department of Clinical Epidemiology, Academic Medical Center, University of Amsterdam, Amsterdam, The Netherlands; 5 Ministry of Health, Kampala, Uganda; 6 Division of Global HIV/AIDS, Center for Global Health, Centers for Disease Control and Prevention (CDC), Atlanta, Georgia, United States of America; University of Oxford, Kenya

## Abstract

In Uganda, men who have sex with men (MSM) are at high risk for HIV. Between May 2008 and February 2009 in Kampala, Uganda, we used respondent driven sampling (RDS) to recruit 295 MSM≥18 years who reported having had sex with another man in the preceding three months. The parent study conducted HIV and STI testing and collected demographic and HIV-related behavioral data through audio computer-assisted self-administered interviews. We conducted a nested qualitative sub-study with 16 men purposively sampled from among the survey participants based on responses to behavioral variables indicating higher risk for HIV infection. Sub-study participants were interviewed face-to-face. Domains of inquiry included sexual orientation, gender identity, condom use, stigma, discrimination, violence and health seeking behavior. Emergent themes included a description of sexual orientation/gender identity categories. All groups of men described conflicting feelings related to their sexual orientation and contextual issues that do not accept same-sex identities or behaviors and non-normative gender presentation. The emerging domains for facilitating condom use included: lack of trust in partner and fear of HIV infection. We discuss themes in the context of social and policy issues surrounding homosexuality and HIV prevention in Uganda that directly affect men's lives, risk and health-promoting behaviors.

## Introduction

Human immunodeficiency virus (HIV) and same sex behavior among men have been associated since very early in the HIV epidemic[1], yet male homosexual behavior has been little studied in Africa due to restrictive policies and substantial stigma and discrimination. Though same sex behavior has been studied in Africa since the 17^th^century [2], most HIV-related behavioral research among men who have sex with men (MSM) has been conducted in North America, Europe and Asia. As more studies measure HIV infection in sub-Saharan Africa, it is becoming increasingly clear that MSM have a significantly greater disease burden than men in the general population [1], [3]–[9].

Risky sexual behavior in other studies in Africa has been associated with having trust in their sexual partners [10], having partners younger than 18 years, and not having had exposure to an HIV prevention program focused on MSM [11]. A research report on MSM risk in Mombasa, Nairobi and Dakar, described inconsistent condom use and multiple concurrent partners as normative behaviors [12]. One study found that men who are bisexual are more likely to use condoms than those who identify as homosexual [13]. Higher risk behavior has also been associated with depression and lower self-efficacy scores among MSM in South Africa[14].

Recently, research in sub-Saharan Africa has consistently reported high levels of stigma, discrimination and physical abuse, with even higher levels reported among certain sub-groups within MSM, such as men who conduct sex work, or identify as transgender[12], [15]–[17]. Perceived stigma has played a role in health seeking behavior where MSM are less likely to seek health care and have difficulty disclosing their sexual orientation to health providers[18].

In Uganda, as in many countries of sub-Saharan Africa, the law criminalizes same sex behavior (Section 140, 141 and 143 Penal Code) and thus makes it particularly difficult for the Ministry of Health to promote prevention or care programs and for MSM to access these services. Though there has been limited research on MSM in Uganda, in 2007 Kajubi et al. conducted a respondent driven sampling(RDS) survey among 228 MSM in Kampala and found that MSM in Kampala are a heterogeneous, highly marginalized and stigmatized group, had high risk behavior and low risk perception [5], [6].

In 2008/9, the Crane Survey, our parent study, was the first study to test key populations at increased risk for HIV and other STIs through an RDS survey of five suspected high risk groups, including MSM [19]. Full description on the Crane Survey including sampling methods and findings related to each population, have been described previously [19]. Briefly, with respect to MSM, survey participants were recruited using RDS [20]. Inclusion criteria were male sex, age ≥18 years, residence in Kampala, and self-reported anal sex with another man in the preceding 3 months. Demographic and HIV-related behavioral data were collected through audio computer-assisted self-administered interviews. The Crane survey found that MSM in Kampala are at markedly higher risk for HIV than the general adult male population and that MSM reporting a lifetime history of homophobic abuse are at increased risk of being HIV infected[19]. Population estimates were adjusted for the non-random sampling frame using RDSAT and STATA. The median age of 300 MSM was 25 years. Overall HIV prevalence was 13.7% (95% confidence interval [CI] 7.9%–20.1%), and was higher among MSM> = 25 years (22.4%) than among MSM aged 18–24 years (3.9%, odds ratio [OR] 5.69, 95% CI 2.02–16.02). In multivariate analysis, MSM> = 25 years (adjusted OR [aOR] 4.32, 95% CI 1.33–13.98. In conjunction with the MSM survey, we conducted a nested qualitative study to explore the social and behavioral contexts of HIV risk, gender identity, stigma and violence with a sub-set of 16 survey participants who reported higher-risk sexual behaviors in their survey responses.

## Methods

### Design, sampling and recruitment

We conducted a qualitative, nested, sub-study of 16 MSM who participated in a cross-sectional respondent driven sampling (RDS) survey, in Kampala, Uganda. These men participated in in-depth interviews to document their perceptions and disclosure of sexual orientation and gender identity, sexual practice, stigma, discrimination and violence, intentions and behaviors relating to risk, prevention and care seeking around HIV and STIs.

A theoretic sampling strategy was used [21] to establish eligibility criteria for the sub-study that would purposively yield a sample of higher-risk MSM across a range of self-defined sexual identity categories. High-risk status was determined through their responses to structured questions in audio computer-assisted self-interview (ACASI) about their sexual identity, sexual activity (inconsistent condom use and/or multiple partners) and other high-risk activity such as drug and alcohol use. Survey participants completed interviews using computers with touch screens employing ACASI, and had HIV infection assessed through serologic testing. At the end of their ACASI interview, participants meeting eligibility criteria were invited to participate in the sub-study and offered an appointment to come back for a face-to-face interview. All participants who reported high risk behavior were offered interviews and accepted. Of note, HIV status was not a selection criteria for the sub-study. All HIV test results were returned to Study participants at a subsequent follow-up visit. Those who participated in the sub-study received their HIV result and post-test counseling at the conclusion of their interview.

### Data collection and analysis

From May 2008 to February 2009, four interviewers (two male, two female) conducted in-depth interviews with participants in the local language of Luganda or in English, as the participant requested, following a semi-structured interview guide. Interviewers were university-trained social scientists who had additional in-depth training for the study including: rapport building, interviewing techniques and use of the qualitative interview guide. Numerous discussions were held before and during the study regarding the sensitive nature of homosexuality in Uganda and how that may affect the research as well as the safety of staff and participants.

Interviews took place in private interview rooms in the Study office. In order to protect the confidentiality and safety of participants, the study office itself was located in an accessible area of central Kampala and was not labeled, and therefore not identifiable to the community as a place frequented specifically by MSM. Participants were asked for their written informed consent, including an explicit consent for tape recording interviews.

All interviews were transcribed and translated into English when necessary. An analysis team consisting of the four social scientists who interviewed participants and an additional senior social scientist coded each transcript. Guidelines that included three distinct stages (sampling and design, theme development, and theme validation and code use) were used for thematic coding as the primary analytic strategy with an emphasis on descriptive thematic coding[22]. After reading two transcripts, the analysis team members collaboratively developed a codebook of themes based on the interview topics as well as those emerging from the data. Two more transcripts were then reviewed to include additional topic areas and themes. This process was repeated until a sample of 12 transcripts had been reviewed and the codebook reached a stage where no new themes or topic areas emerged. To ensure inter-rater consistency, the analysis team compared their individual coding of the same transcripts. All transcripts were then coded using the final version of the codebook and merged using NVivo software (version 2.0, QSR International Pty. Ltd, Victoria, Australia) before themes were summarized across respondents. After coding, the merged project was transferred to NVivo version 8 for analysis. Analysis focused on identifying the dominant and the range of explanations for sexual identity, sexual behavior, motivations for preventive behaviors and comparisons across clients. Multiple (monthly during analysis) interactive discussions were held with the analysis team and senior researchers to validate data interpretations and resolve any interpretation discrepancies.

The study was approved by the Institutional Review Boards of the Uganda National Council on Science and Technology, Entebbe, Uganda, and the Uganda Virus Research Institute, Entebbe, Uganda, and was reviewed and approved by the ADS Office of the Centers for Disease Control and Prevention, Atlanta, Georgia, USA. All clients provided written informed consent for participating both in the main survey and separately for the qualitative interview.

## Results

Demographics have been described [19] in the larger sample from which our sub-study were included (Table 1). Briefly, half the men in our sample were over 24 years, over three quarters had seven or more years of education, 31% were HIV infected, 100% reported ever drinking alcohol.

**Table 1:** Participant Characteristics in the Quantitative and Qualitative Studies, 2009, Uganda.

Characteristic		Total Quantitative Sample un-weighted N = 295(%)	Total Quantitative Sample weighted N = 295 (%)	Total Qualitative N = 16 (%)
Age	18–24 years	143 (49)	138 (50)	8 (50.0)
	25+ years	152 (51)	143 (50)	8 (50.0)
Years in school	6 or less years	71 (25)	68 (25)	2 (12.5)
	7+ years	218 (75)	205 (75)	14 (87.5)
Occupation	Student	52 (17.8)	49 (18.5)	4 (26.7)
	Unemployed	49 (16.8)	48 (16.0)	2 (13.3)
	Employed	191 (65.4)	181 (65.5)	9 (60.0)
HIV Sero-status	Negative	254 (86.4)	244 (86.3)	11 (68.7)
	Positive	40 (13.6)	36 (13.7)	5 (31.3)
Ever drink alcohol	Yes	61 (20.8)	217 (79.6)	16 (100)
Sex orientation	Gay/Homosexual	166 (56.9)	158 (55.0)	11 (68.7)
	Bisexual	113 (38.7)	107 (37.6)	5 (31.3)
	Straight/Heterosexual	13 (4.4)	13 (7.4)	-
Gender Identification	Male	209 (70.9)	203 (75.8)	9 (56.2)
	Female	52 (17.6)	52 (20.5)	3 (18.8)
	Neither	13 (4.4)	13 (3.7)	-
	Missing	21 (7.1)	13 (0.0)	4 (25.0)
Ever fathered children	Yes	94 (32.2)	89 (29.6)	3 (18.8)
Ever disclosed sex with men	Yes	175 (59.3)	166 (55.3)	11 (68.8)
	No	110 (37.3)	105 (41.5)	4 (25.0)
	Skipped	10 (3.4)	10 (3.2)	1 (6.2)
Ever used a condom	Yes	208 (70.5)	196 (70.3)	13 (81.3)
	No	76 (25.8)	75 (26.4)	1 (6.2)
	Skipped	10 (3.4)	10 (3.3)	2 (6.2)
	Don't know	1 (0.3)	0 (0.0)	3 (6.2)
Ever paid for sex	Yes	123 (43.3)	115 (35.2)	5 (35.7)
	No	142 (50.0)	132 (56.9)	7 (50.0)
	Don't remember	9 (3.2)	7 (4.9)	1 (7.1)
	Skipped	10 (3.5)	10 (3.0)	1 (7.1)
Ever sold sex	Yes	129 (43.9)	120 (38.8)	11 (68.8)
	No	155 (52.7)	150 (58.1)	4 (25.0)
	Skipped	10 (3.4)	10 (3.0)	1 (6.2)
Experienced violence/abuse due to MSM activity	Yes	115 (39.4)	106 (35.1)	8 (50.0)
	No	167 (57.2)	160 (61.7)	7 (43.8)
	Skipped	10 (3.4)	10 (3.2)	1 (6.2)

### Sexual orientation and gender identity

Though sexual orientation and gender identity in this context presents as a dynamic continuum, for this analysis we have categorized emerging data into three main self-identification categories as: “Gay” (men who had sex exclusively with other men), “bisexual” (men who have sex with both men and women), and “transgender” (individuals who self-identified as “women” and who have sex with men). None of the qualitative participants self-identified as heterosexual. Each category is grounded in the meanings as explained by participants themselves in the local context and all are described in detail below.

#### Gay

There were ten men reporting as gay with five of them also identifying using language that could be defined as transgender. The following man asserted that he first slept with a woman and did not feel comfortable, “*I realized that when I slept with a man, I was very comfortable and I was free* …” (>24 years old, HIV-negative).

One man who had fathered a child stated that he used to feel bisexual but now believes he was born gay because his cousin is also gay and he believes there is a genetic component to sexual orientation. He also explained that he feels more at peace with his sexuality, “*I feel complete. I have no regrets about this thing because that is my nature. I was born like that I feel good because it is how I was born*” (<25 years old, HIV-positive).

#### Bisexual

Six men self-identified as bisexual, and each one described bisexual identity differently. One man appeared to feel equally attracted to both men and women. He said,

*Hmmm, at times I mix. I have women, I have men. So, no difference. It's not different*

(<25 years, HIV-negative).

Similarly, a 28-year-old man, stated that he was bisexual because he has sex with both men and women and would not feel comfortable marrying either one. He has two children from different women, but does not stay attracted to women for very long despite saying that many women were attracted to him.

Another man described how his attraction to women was changing and over time he was becoming more attracted to men, but he felt conflicted due to the strong cultural bias against homosexuality.

*Because I still have feelings for ladies, for women. I don't feel man enough of course. I don't feel like, (Silence)*

*[…] I don't want anyone to find out […] It is abnormal to them. Me, I feel I am not a man. I am not man enough, how does a man sleep with a man? […]*(>24 years old, HIV-positive).

The following man narrated that he has two permanent partners; one man and one woman.

*I feel proud of my feelings, even what am doing […] Yeah it is normal* (>24 years old, HIV-negative).

#### Transgender/“I feel I am a woman”

In our qualitative sample five individuals described themselves as “*born to be women*”. For purposes of coding, “transgender” was derived from the American Psychological Association definition: “transgender is an umbrella term used to describe people whose gender identity or gender expression differs from that usually associated with their birth sex”[23]. These individuals qualified that they identify as transgender or as women because of how they feel and act in their daily lives, because of who they are attracted to and how they relate to other men and women. When these individuals were depicting their gender identity most appeared at ease with it despite the restrictive policies and social norms in Uganda.

*I was born with that orientation even when I was young I was always with women […] Even the strength in the body is feminine I feel different in the body and that is how God wished. I think I can look after a man better than a woman- I am searching for the day I get one he will believe that he has the best* (<25years old, HIV-negative).

Interestingly the statement below portrays the complexity of feeling at ease with oneself in spite of a double stigmatization. The following participant compares herself to male-identified gay men and how she feels more stigmatized as transgender.

*[…] but my sex orientation can prove what I am, I am transgender and I am a woman. That is why I always tell people that they should not call me a man; they should call me a woman because I know I am a woman […] This kind of discrimination is within the main community and in the gay community as well. Some gay men don't want to associate with transgenders. You know some gay men want to hide their identity; they dress like real men and put on coats and ties. Some drive themselves. When he sees that you are a transgender, he will fear to walk with you or be in your company. You know we as transgenders we try to bring out what we really feel we are. But for them they enjoy having sex with men but cannot come out in the community to show their real identity. They tend to hide their real identity […] I feel I am a woman; I have to behave like a woman. Such things create problems for us. Discrimination is very high even among the gay community when it comes to transgenders* (>24 years old, HIV-positive).

Some individuals have gleaned reassurance and self-acceptance from their feelings of being born with their female gender identity. The following participant highlights the distinction between gender identity and sexual orientation.

*I feel happy that I came to accept myself the way I am. In the village […] they may not know that maybe I am gay. But they really know that I am like a woman […] I was born like that* (>24 years old, HIV-negative).

Some participants attribute their gender identity to their religious beliefs. The following statement shows how one participant's belief in god has relieved the responsibility for trying to change her feelings.

*I feel good because I was born like that and I am not interested in being a man. I also thank God for the way he created me. It is not me who made myself; it was God's wish. Should I abuse God who created me like that, what do I do?*(<25 years old, HIV-negative).

Others expressed feelings of isolation related to their gender identity.

*My biggest problem is I am a man but I am a woman […] I think I was born like that and I don't know anybody like me* (>24 years old, HIV-positive).

This individual explained that she has disclosed her gender identity to others and feels comfortable about it because she feels she cannot change who she feels she is.

### Disclosure of sexual identity and sexual orientation

Some individuals expressed having disclosed their sexual orientation to trusted individuals, while most described living double lives.

In the quantitative sample 55% of participants indicated having disclosed their sexual orientation. Individuals who have disclosed have generally done so to people they deem they can trust. In some cases it was friends, in others siblings or parents. Some have disclosed only to other gay men as this was perceived as safe because gay men could be trusted.

*These people are like me because we know we are all gay. So, when I tell them that I am gay obviously they have to be supportive (<25 years old, HIV-negative).*

Similarly, another man narrated that his reason for disclosure was because he wanted to have sex with the man he disclosed to.

Some men have not disclosed to anyone close to them and have devised methods for hiding their behavior. One 26-year-old man stated,

*Actually none of my closest friends know I am gay […]. it is my secret life and Uganda is not a free country*” (>24 years old, HIV-negative).

After describing how he had met many other gay men over the internet and narrating how it is illegal and unsafe saying, ‘*we live with it under covers*’ (>24 years old, HIV-negative). This man describes how he ‘*moves with girls most of the time*’ to hide his sexual identity and avoid questions from friends. Disclosure of sexual orientation appears to be related to men's feelings of self-confidence regarding their sexual identity. Many men who described being comfortable with their sexuality, despite the legal and social issues in Uganda, were able to disclose and those who live in fear have found it much more difficult to disclose. One 24 year old man said, “*Okay, there is no one I have narrated to […] because I can't be proud about it*” (<25 years old, HIV-negative). Another man mentioned that he has not told his girlfriend that he has sex with other men (>24 years old, HIV-negative). However, as one participant explained even though he was with other gay men he still did not feel comfortable disclosing because he felt shame (>24 years old, HIV-negative). Similar to others in this study, this man embraces and accepts his orientation in some situations, yet alludes to a feeling of denial towards his mother and a wider community with regards to disclosure.

*“Yeah I want to look acceptable in my mum's face but indeed I know who I am, I am gay. Yeah, at least I love being me; I don't want to let the whole public know that I am an MSM. Okay people may see me with guys only and they suspect […] but won't be able to exactly know what is going on […] I feel naturally I was born to be [gay] (<25 years old, HIV-negative).*

### Stigma, discrimination, and violence

Our data described enacted societal stigma and discrimination as well as self-stigma and physical violence. Uganda is well known for its restrictive views on homosexuality [24], [25]and participants portrayed the effects in personal terms. One man stated, “*it is not safe here […]. You know we are criticized; the country does not allow it here* (>24 years old, HIV-negative). Another put it as,

*There is no freedom in Uganda; once found you are hated and isolated* (>24 years old, HIV-negative). Another asserted that gay men are treated like animals, “*It is abnormal to our culture […] A man should marry a woman […] when you are walking on the street they throw stones at you […] They say bad words […](Silence). They treat us like, […] Satan. They don't treat us like human beings. They harass very much[…]the way they treat animals. They harass animals. Like cows. They are always beaten* (>24 years old, HIV-negative).

One man, who self-identified as transgender, depicted abuse from family, employers, as well as police.

*I worked with the Catholic Missionaries long ago. When I completed my Senior 4 [*fourth year of secondary school*], I got this job right away. I worked among Catholic priests; most people wanted me to become a Catholic priest. I worked with them and the moment they got to know about me like this, I was asked to leave […] My father sent me away from home because he wanted me to be straight, he sent me away from home to discipline me […] But deep within me I refused to stick to my parents' expectations, that is why I packed my things and left my parents' home*(>24 years old, HIV-positive).

Participants who identify as transgender described even greater discrimination and often feel isolated from the gay community as well as the general society.

*You are like a sacrifice. We are also discriminated against in the gay community, discriminated against by family members. Then you also feel out of place, you feel low esteem. You don't feel comfortable, you are not free. You are not proud. This kind of discrimination is within the main community and in the gay community as well* (>24 years old, HIV-positive).

Half of the interviewees had experienced physical or psychological abuse. Some individuals had been beaten and raped numerous times and have had to devise ways to avoid constant harassment. Stories of abuse have taken place in the family, have occurred in the street, and have been perpetrated by people in positions of power.

*At night, the police also used us […] we reached a certain forest which I don't know the name and the driver said the vehicle had some mechanical problems and this man also raped me, this man just lied because the vehicle never had any mechanical problem […] People raped me and I was feeling a lot of pain. I was repeatedly raped […] I also got many problems in Kampala, I was raped five, no seven times; I have very many problems. Those security personnel, the policemen and others […] they abuse me, but what can I do, that is the way I was created […] Yes, they beat me, the boda men [*motorcycle taxi*] beat me, the chairman [local leader] beat me, they don't want me to enter taxis[…]* (<25 years old, HIV-negative).

One HIV infected man described abuse by the police as follows:

*[…] the police would interrogate you and scare you off, but after they come back and make advances. They do it under the pretense of arresting you when he actually loves you. He takes you somewhere and he molests you* (>24 years old, HIV-positive).

One of the most commonly described forms of discrimination MSM experienced was in health care settings. Participants described different strategies to cope with discrimination at health facilities. Some devised means to use health services by seeking a gay provider, or asking friends to play a ‘go-between’ role. Others said that the best means is self-treatment because they felt they would not be treated at health facilities anyway.

*I always go to hospitals and they easily tell that I am gay. I ask for condoms but usually a health worker will tell you to sit down and wait. Then he calls his co-workers, they peep through a window and laugh/mock you. This makes me feel very bad. So, I find it easier to use my friends to pick up condoms for me. Sometimes, I just go straight and buy them instead of getting them for free from hospitals* (>24 years old, *HIV-positive*).

*Even if I fall sick or get fever, I just stay home without treatment because you can't go to the main referral hospital in Kampala. There, every health worker will object to giving you treatment saying that “he is a homosexual don't work on him” and say many other things. I was told that very many times, about six or eight times. Like when I was assaulted, don't you see here at the ear, there is (Embunda; scar/wounds) […] they neglected and chased me away and I was bleeding and swollen. I came back home and slept and got healed by God's mercy (<25 years old, HIV-negative).*

Health provider discrimination combined with self-stigma results in participants often not trying to access treatment. The following transgender participant stated that:

*When you go to visit the hospital, they will not attend to you. In fact I hate going to such hospitals. I do self-treatment from home and I usually use tablets. You know I feel ashamed. I will visit the hospital and everybody will despise me. It is the way female health workers treat me, they make me feel angry and resentful to seek treatment. That makes me feel ashamed. Everybody looks at you. You feel you are not part of the society* (>24 years old, *HIV-positive*).

A few individuals mentioned that they knew of a gay doctor who could be trusted to consult with at one of the hospitals in Kampala.

*I came to know recently and maybe if we went to […] hospital where Dr XX was working. Maybe they could be easily attended to because he would send them to officers who could treat them with care […] he is gay* (>24 years old, HIV-negative).

### Coping Strategies

To cope, participants described various strategies to hide their relations with other men. Some said that they have initiated relationships with women as a disguise, masqueraded as straight men, some mentioned that they changed residence frequently so that people who might harass them would not be able to find them. An additional tactic one man mentioned was to befriend anyone who tried to hassle him. One bisexual man said that he never gives his telephone number to anyone.

*I try not to call anyone. I make sure they don't call me too. And for that, I am very strict. I make sure Hmmm […] I don't show them where I stay. I always associate with gentlemen […] We try to hide it. Try to make sure that we still look like men* (>24 years old, *HIV-negative*).

The following man described the great extent he is willing to go to lead a double life.

*Marrying a woman is also demanding, money demanding. Eee! Yeah, you get into the marriage project when you are ready to see it to the end. Hmmm […] So, actually I have a friend […] Okay, she is like a friend but I know she has feelings too and truly loves me […] But I don't know because if I am to tell her (*that I'm gay*) […] I will [get married] just to please my mum but it is going to cost me a lot […] But this is someone who loves swimming and clubbing. So you have to make her feel comfortable with all that and – right now, I am just looking for someone, just to “kusibakiwaani”* [disguise with] (<25 years, HIV-negative).

To cope with the potential for violence associated with sexual behavior some men noted that they had to relocate frequently.

### Condom use

Because men in this sample were purposively recruited on the basis of factors associated with higher risk for HIV infection, discussions of condom use focused primarily on barriers to consistent condom use. Participants identified three broad themes: accessibility, negative attitudes towards condom use, and trust between partners.

Two men stated that condom use depends on how one categorizes his partners either casual or more serious.

*No, for the temporary ones (*partners*), it is a must to put on a condom whether you want to or not (<25 years, HIV-positive).*

Having tested for HIV and found positive, this man described his methods for ensuring condom use;

*‘And I don't have money, I just call some of my friends and ask them to buy for me some condoms. This I do it earlier if I expect to get a sex partner within the coming two days. I really struggle to have some condoms which I always keep at home. I ensure that I do not run out of condoms* (>24 years, HIV-positive).

This same man, however, has also described situations where he did not use condoms, associated with alcohol use, and on occasions when he had sex with trusted partners.

Some of the factors influencing the use of condoms include lack of trust or discomfort with condoms. More than one man mentioned that when he has not seen a partner for a long time he will insist on condom use.

*Lack of trust, that is one factor which at times makes me use condoms […]* (<25 years, HIV-negative).

This same man mentioned later in the interview that his partner complained that the condom was too small among other issues,

*[…] he says that it is tiresome […] it is not interesting […]* (<25 years, HIV-negative).

Later still in the interview, he said,

*We may use, we may use it around eight times or ten times, you say aah [meaning “I have had enough”]. Since we have known each other […] It is now four years and […] let us trust each other[*meaning that they will stop using condoms*]* (<25 years, HIV-negative).

One man, who described himself as a sex worker, consistently uses condoms with his male clients, but inconsistently uses them with his girlfriends. With women he stated, ‘*At times we use, at times we don't.*’ He noted that he knows the HIV status of the girlfriend that he loves as she tested when she was pregnant (>24 years, HIV-negative). Another man noted that it's a matter of trust; for partners he trusts, he doesn't insist on condom use.

*There are times when I don't use condoms. Usually it is with boys of my age […]*(>24 years, *HIV-negative*).

Not having enough money to purchase condoms was a factor in influencing their use as well.

*Okay, you know condoms when you use them they even break. If it had broken, do you stop with sex? You go live. You need to have money to buy condoms* (>24 years, HIV-positive).

## Discussion

This is the first qualitative study of MSM in Uganda that we are aware of. Our findings of 16 in-depth interviews of MSM in Kampala reveal that men self-identify as gay, bisexual and transgender despite the restrictive policy and cultural environment in Uganda. All participants described personal or vicarious experiences of stigma, discrimination and violence in relation to their sexual orientation and have been affected personally in some way. These findings are similar to other studies across Africa where MSM report high levels of sexual victimization, physical and verbal abuse[12]. In our study, individuals who identify as transgender described greater perceived stigma and discrimination than men who identified as gay; other studies suggest they also may be at increased risk for infection [17].Greater stigma and discrimination may be due to their appearance and lack of secrecy. Men describe experiences where they are ostracized from their families, dismissed from places of employment, thrown out of schools, severely discriminated against in health care settings and beaten by police for their sexual orientation. Our participants were provided with counseling following the in-depth interview, but more targeted services and a shift in the legal framework would potentially limit the increased stress from social isolation and limited access to health services.

Our findings on inconsistent condom use among MSM are consistent with ethnographic and survey findings throughout the Sub-Saharan Africa region. [7], [15], [26]. As has been found among heterosexual populations, assumptions of trust between partners serves as a powerful disincentive to condom use. Okal and colleagues noted among MSM in Kenya that levels of condom use decreased with degree of intimacy and stability of relationships [15]. Confronting prevalent beliefs that trusting a partner is equivalent to having the same HIV status could play a significant role in mitigating risk behavior. The only way to address these beliefs is to promote disclosure of HIV test results or couple testing. A recent study however found that communicating HIV results was not associated with practicing safer sex [27]. Condom accessibility and desirability were important factors in limiting condom use in our study; thus attempting successful strategies used by others in similar situations might increase uptake. Possible recommendations could include: greater open discussion with health care providers to limit homophobia and stigma, increased HIV testing, development of evidence-based individual-level, couple-based and group-level risk reduction interventions, leadership from the gay community, use of peers, opening up avenues for free condom distribution outside of formal health care settings and recognition of personal action [7], [28], [29].

Our study is not without limitations. This was a higher-risk sample by design and therefore it is not possible to generalize these results to an entire population of MSM in Uganda. In addition, our study was conducted only in Kampala, so would not be generalizable to rural areas. However, these data refer to the in-depth experiences of individuals across a range of sexual orientation/gender identity groups, including transgenders, who are at highest risk of HIV infection. The study however has important implications as the first qualitative exploration of MSM in Uganda.

In reflecting on how these findings relate to potential for intervention, we found that social isolation and family ostracism force many men to keep their sexual identity a secret from friends as well as health workers [15] thus any intervention must be designed to accommodate individuals who have been hiding, keeping phone numbers a secret, exhibiting high mobility such as frequently changing residence, and trusting few individuals. Men develop intricate strategies for coping with discrimination and interventions must recognize and respect these. For example, condom distribution within health facilities would not likely benefit MSM in Uganda as few health facilities are welcoming environments for this population. Some men in our study mentioned the use of internet to find other MSM (data not reported here); thus internet or mobile phone use might be a more safe and effective mechanism for MSM-specific health information. Community outreach strategies could prove to be an effective mechanism for transmitting HIV prevention messages considering communication channels most frequently discussed in our study population. Other interventions, such as the Health4Men project in South Africa have designed services to specifically address MSM issues such as homophobia, stigma and discrimination in relation to HIV risk and service delivery[30].

MSM are still an “understudied and underserved” group [1], [12] in Africa due to the political criminalization and social stigmatization. Prevention and care interventions must be designed with the social and political contexts in the foreground. Structural interventions will have to be considered; though weighing the feasibility vs. effectiveness of such interventions will constantly be challenging. Human rights perspectives in this situation are paramount; MSM rights in Uganda are not protected by the state (as in 30 other sub-Saharan African countries[7]), thus any prevention or care services must first consider the safety of the target group before the coverage of its services. HIV prevention programs will not successfully target MSM until they can feel safe from stigma, violence and discrimination.
